# Scientific Evaluation of the Court Evidence Submitted to the 2019 Human Papillomavirus Vaccine Libel Case and Its Decision in Japan

**DOI:** 10.3389/fmed.2020.00377

**Published:** 2020-07-29

**Authors:** Jason M. Bodily, Ikuo Tsunoda, J. Steven Alexander

**Affiliations:** ^1^Department of Microbiology and Immunology, Louisiana State University Health Sciences Center - Shreveport, Shreveport, LA, United States; ^2^Center for Molecular and Tumor Virology, Louisiana State University Health Sciences Center - Shreveport, Shreveport, LA, United States; ^3^Department of Microbiology, Kindai University Faculty of Medicine, Osakasayama, Japan; ^4^Department of Molecular and Cellular Physiology, Louisiana State University Health Sciences Center - Shreveport, Shreveport, LA, United States

**Keywords:** animal model, anti-vaccination, anti-vax, HANS, HPV vaccination associated neuro-immunopathic syndrome, vaccine hesitancy, viral model

## Introduction

Human papillomavirus (HPV) infects the skin and other body surfaces causing warts and other benign growths (1). Although most HPV infections are eliminated by the immune system without complications, some HPV-induced growths can progress to cancer. HPV-induced cancers, including cervical cancer and oropharyngeal cancer, are responsible for over 300,000 deaths annually worldwide (2), making HPV infection a major public health problem. Several HPV vaccines have been shown to safely and effectively prevent infection by cancer-causing HPV types, thus preventing the antecedent growths that inevitably lead to cervical cancer (3, 4).

In Japan, HPV vaccines were initially introduced in 2011, and became routinely used in 2013 when the vaccination rate approached ~70%; however, after only two and a half months, the Japanese government suspended proactive recommendation of HPV vaccination. The suspension was based on clinical reports of suspected adverse events from a few girls after HPV vaccination. Testimonials from these girls and medical doctors in Japan were repeatedly broadcasted on TV, creating public fear of the vaccine which prompted withdrawal of government support (5).

## HPV Vaccine Libel Case

Public anxiety over HPV vaccination was amplified by the experimental findings that were presented to the Ministry of Health, Labor and Welfare (MHLW) of the Japanese government, on March 16, 2016 by Dr. Shuichi Ikeda, principal investigator of the research team funded by MHLW who investigated potential nerve injury following HPV vaccination. In July 2016, a class-action lawsuit against the Japanese government asking for compensation for the damage purportedly caused by the HPV vaccine was filed; this lawsuit is still ongoing. The “temporary” suspension of the proactive recommendation for the HPV vaccines will have been in effect for 7 years as of June, 2020. Although evidence for the safety of this vaccine has been recognized internationally (6, 7), the HPV vaccination rate in Japan remains below 1%, thus placing coming generations of young Japanese women at unnecessary risk of cervical cancer in the future. In 2017, the Global Advisory Committee on Vaccine Safety (GACVS) reported that the mortality rate from cervical cancer in Japan increased 3.4% between 1995 and 2005 and was understood to increase by 5.9% between 2005 and 2015 (8). Recently, Simms et al. estimated that Japan's termination of HPV vaccinations will result in 5,000 deaths due to cervical cancer (9).

The summary of the MHLW presentation by Dr. Ikeda was broadcast on the evening news show, “NEWS23” on Tokyo Broadcasting System (TBS) Television network on March 16, 2016. In that news show, Dr. Ikeda, Professor and Dean of Shinshu University, Nagano, Japan, purported to show experimental evidence of brain damage in a mouse injected with an HPV vaccine (https://www.mamoreruinochi.com/wordpress/wp-content/uploads/docs/publication/hei79-1.mp4?fbclid=IwAR3F48afJCKFXyrppMtWQhDQqo3T6BdQWSLRZV7DuWcrl2fuH3Yiz9JPc0Q). Dr. Ikeda explained, “Deposition of abnormal antibody was observed only in the brain section of the mice injected with the HPV vaccine. The function of the hippocampus seems to be damaged. Apparently, the brain is damaged.” This broadcast influenced public opinion and helped raise an alarm against HPV vaccination. Two weeks after the show, the HPV vaccine “victims” announced they were suing both the government and the vaccine manufacturers.

In June 2016, in response to Dr. Ikeda's team presentation and broadcast, Dr. Riko Muranaka, a noted physician, journalist, and vaccine advocate, wrote in the business magazine *Wedge* that Dr. Ikeda's experimental results suffered from significant scientific irregularities, and suggested that they may have been fabricated. In response, Dr. Ikeda sued Dr. Muranaka for libel, that she had damaged his reputation as a scientist. On March 26, 2019, a court in Tokyo found Dr. Muranaka guilty of libel since the Tokyo district court could not find evidence that Dr. Ikeda intentionally engaged in scientific misconduct. On October 30, 2019, the Tokyo High Court ruled against Dr. Muranaka in a retrial of the defamation case, despite support for Dr. Muranaka from Dr. Tasuku Honjo, a Nobel laureate. Since an appeal to the Supreme Court of Japan was dismissed on March 9, 2020, the above judgement became final.

However, a critical point about this court decision was that it was based solely on the impact of Dr. Muranaka's usage of the word “fabrication” to harm Dr. Ikeda's reputation. The decision did not asses the scientific accuracy of the experimental findings. Therefore, although the result of the trial can be (and has been) seen as a victory for anti-vaccination advocates, the actual safety of the HPV vaccine was not at issue. Although the trial was reported in several prestigious journals in English including *Nature* and *Science* (10, 11), little scientific information about the evidence used in the trial has been available in English. Because of the importance of this trial in worldwide efforts to promote HPV vaccination, we summarize the scientific evidence submitted to the trial and during Dr. Ikeda's broadcast to assess its scientific merit using our professional expertise in virology, immunology, and neuroscience.

Because the scientific accuracy of Dr. Ikeda's team data was unclear, Shinshu University formed a committee to investigate the experimental findings. Dr. Ikeda's team showed images of antibody-induced damage in the hippocampus following HPV vaccination in “a mouse” (or “mice”; in the Japanese language, a singular form is commonly used instead of a plural form in most occasions). The research team claimed that they could not find such damage in mice injected with hepatitis B virus vaccine, influenza virus vaccine or phosphate-buffered saline (shown in a Japanese slide at https://www.mamoreruinochi.com/wordpress/wp-content/uploads/docs/publication/kou07.pdf and Slide 31 in English at https://www.mamoreruinochi.com/wordpress/wp-content/uploads/docs/publication/kou17.pdf). However, it turned out that the hippocampal picture was not from a vaccinated mouse (as Dr. Ikeda said in the TV broadcast) but from a brain section from an *unvaccinated* mouse onto which sera collected from vaccine-injected mice were applied. Even worse, although the experiment should have been conducted using multiple mice for accuracy, Dr. Ikeda's team used serum from only a single mouse. Dr. Ikeda's presentation neglected to share this important fact. Moreover, the vaccine-injected mice were not normal mice, but rather mutant mice that are known to have abnormal antibody production (12); immunologically, normal mice should have been used in the experiment since the mutant mice could develop abnormal autoantibody production even without treatment. Under the supervision of the Shinshu University committee, the same experiment was repeated by the same research group. The second experiment demonstrated no antibody deposition on hippocampal sections, which were incubated with sera from three HPV vaccine-injected mice or three control mice; negative results from both HPV vaccine and control groups were shown in the third slide at https://www.mamoreruinochi.com/wordpress/wp-content/uploads/docs/publication/hei15.pdf).

## Discussion

There is a consensus in the Japanese scientific community, including the Shinshu University committee and MHLW of the Japanese government, that Dr. Ikeda's research team did not prove that HPV vaccination caused damage in mouse brains. As virologists, immunologists, and neurologists, we evaluated the evidence submitted to the trial and fully agree with that consensus. The “finding” presented by Dr. Ikeda that the HPV vaccine causes hippocampal damage was not supported by subsequent work, even performed in the same University by the same group. No credible scientist can accept the possibility of the “adverse effect” of an HPV vaccine from a single mouse hippocampal image, especially when the total number of the mice used in the experiment was withheld. As of today, Dr. Ikeda's team has neither published a manuscript on the effect of HPV vaccine using valid experimental design nor disclosed the number of mice used in the experiments broadcasted on TV. Another Japanese group published a manuscript on an animal model for HPV vaccination associated neuro-immunopathic syndrome (HANS) in a journal, *Scientific Reports* in 2016, but the publisher retracted the article because the experimental approach did not support the conclusions of the study (13). Therefore, experimentally, there is no evidence that the HPV vaccine can induce brain damage.

In evaluating the significance of the recent trial, it is critical to separate the legal issue of libel from the scientific issue of evidence. The evidence indicates that Dr. Ikeda's teamwork says nothing about the safety of the vaccine. Even if Dr. Ikeda's research team did not fabricate the data that he presented, the data clearly did not support his claims and therefore his research team's “findings” do not support the notion that the vaccine is in any way dangerous. We fear that the publicity of this trial will further damage the reputation of the HPV vaccine in Japan, and perhaps worldwide. We hope that careful consideration of the evidence and of the issues involved will help put to rest some of the concerns and fears of the public and thus remove some of the barriers to this important vaccine.

## Author Contributions

JB and IT researched the topic and prepared the text. JA supervised and edited the text and provided feedback. All authors reviewed the manuscript and agreed with the decision to submit for publication.

## Conflict of Interest

The authors declare that the research was conducted in the absence of any commercial or financial relationships that could be construed as a potential conflict of interest.
